# Introducing the Prototypical Stimulus Characteristics Toolbox: Protosc

**DOI:** 10.3758/s13428-021-01737-9

**Published:** 2021-12-16

**Authors:** S. M. Stuit, C. L. E. Paffen, S. Van der Stigchel

**Affiliations:** Department of Experimental Psychology, Utrecht University, Utrecht, Netherlands

**Keywords:** Image Statistics, Machine Learning, Toolbox, Matlab

## Abstract

Many studies use different categories of images to define their conditions. Since any difference between these categories is a valid candidate to explain category-related behavioral differences, knowledge about the objective image differences between categories is crucial for the interpretation of the behaviors. However, natural images vary in many image features and not every feature is equally important in describing the differences between the categories. Here, we provide a methodological approach to find as many of the image features as possible, using machine learning performance as a tool, that have predictive value over the category the images belong to. In other words, we describe a means to find the features of a group of images by which the categories can be objectively and quantitatively defined. Note that we are not aiming to provide a means for the best possible decoding performance; instead, our aim is to uncover prototypical characteristics of the categories. To facilitate the use of this method, we offer an open-source, MATLAB-based toolbox that performs such an analysis and aids the user in visualizing the features of relevance. We first applied the toolbox to a mock data set with a ground truth to show the sensitivity of the approach. Next, we applied the toolbox to a set of natural images as a more practical example.

**Supplementary Information:**

The online version contains supplementary material available at 10.3758/s13428-021-01737-9.

## Introduction

Many types of experiments compare behavioral or neurophysiological responses to variations of visual stimuli. With all else being equal, the variations in the stimuli are then considered the source of any possible difference between these responses. When, for example, two conditions consist of differently oriented Gabors, interpretation is not that difficult since these conditions will only differ along one dimension: orientation. However, when using natural or other forms of complex images, these may differ on many known and unknown dimensions, including local and global contrast, local and global orientation, luminance, color, and so on. In fact, many studies have demonstrated image property differences as confounding factors when interpreting results (see the following for example: Stein, Awad, Gayet, & Peelen et al., 2018; Gayet et al., 2019; Purcell et al., 1996; Purcell & Stewart, 2010; Savage et al., 2013; Savage & Lipp, 2015; Moors, Boelens, van Overwalle & Wagemans, Moors, Boelens, et al., 2016a; Moors, Wagemans, & de-Wit, L., 2016b; Gelbard-Sagiv, Faivre, Mudrik & Koch, Gelbard-Sagiv et al., 2016; Heyman & Moors, 2014; Willenbockel et al., 2010). One way to deal with this potential problem is to equate the stimulus content of categories of images as much as possible (see Willenbockel et al., 2010 for a toolbox that does just that). However, since such an approach changes the image content in a way that alters the appearance of the images, another approach is to quantify the image differences with the aim of finding the source of any possible difference in the responses to them. The goal of Protosc is to provide an easy-to-use means to identify and describe the differences in the stimuli that have predictive value of their category and may relate to and/or cause differences in behavioral and neurophysiological responses to different image categories, which does not require previous knowledge of feature selection and machine learning.

Protosc automates the extraction of features from images reflecting either the Fourier magnitude spectrum, Fourier phase spectrum, Histograms of Oriented Gradients (HOG), color value distributions, pixel intensities or a combination of these. Note that HOG and pixel intensity features are highly spatially specific and the Fourier phase spectrum to a lesser degree so, while the Fourier magnitude spectrum and color value distributions are position-invariant. The feature space of interest can be exploratory or hypothesis-driven. For example, Campbell and Robson (1968) showed that contrast sensitivity is a function of spatial frequency: contrast detection sensitivity showed a peak sensitivity around 1 degree per visual angle, with sensitivity dropping for both higher and lower spatial frequencies. Furthermore, in 1972, Appelle showed that discrimination sensitivity is greater for cardinal orientations than for oblique orientations, a sensitivity difference referred to as the “oblique effect”. As such, more contrast energy around 1 degree per visual angle or for cardinal orientation in one category of images compared to another may bias the results due to a sensitivity difference. To provide insight into the relevant differences between categories, our approach focuses on machine learning-based feature selection via multiple methods simultaneously. Specifically, while two of the four methods for selection aim to find relevant combinations of features by finding the most probable features of the images that most likely lead to high cross-validated performance, the other two methods select features at random. The benefit of the random selection methods is that they provide insight into the degree to which the image categories differ. In other words, high performance based on randomly selected features suggests extensive differences between the categories. Via this route, we aim to provide a more complete view of differences between the categories without testing every possible combination of features. Note that the classification models used in this toolbox do not include deep learning models. This is because deep learning models will, by definition, create their own features (Deng & Yu, 2014), and we aimed to keep the features as easy to interpret by the user as possible.

Here, we present a toolbox for automatically analyzing the image content of two or more image categories. Our approach uses feature selection and machine learning algorithms to objectively find features of the images that have predictive value over the predetermined categories the images belong to. This approach ranks image features using the performance of classification models, meaning the interactions between the different features that help to separate categories will also be taken into account. The main goal is to allow insight into how the categories differ. However, the significant features can be taken into account when interpreting behavioral differences based on the used categories (i.e. as covariates). This toolbox is implemented in a MATLAB environment. We will start by focusing on the main approaches used in the toolbox, namely, image processing and feature selection based on classification performance. Next, we will demonstrate the sensitivity of the approach. Finally, we will highlight the usability of the toolkit.

## Methods

### Terminology

**Category or Class:** A set of images that are grouped together by a user. For example, a set of images of angry faces.

**Feature Space:** A qualitative description of an image. For example, the image can be described in terms of its spatial frequency contents.

**Feature:** A quantitative or numeric description of an image in a subpart of a feature space. For example, a specific orientation in a specific location of the image (this would be an example of a feature of the HOG feature space).

**Example:** A description of a single image in terms of a label and all its feature scores.

**Label:** Numerical indication of the category an example belongs to.

**Set:** Collection of individual examples of images consisting of scores on all features and a label.

**Fold:** A fold refers to one out of *k* steps of *k*-fold cross-validation. There, the data are separated into training data and test data *k* times such that every example is part of the test data once. The test data are often referred to as the holdout data, since they are kept separate from the training data to avoid a model being trained with prior knowledge of the data on which it will be used to decode.

### Protosc version

The following methods are based on version 1.03 of Protosc, which can downloaded from the Protosc Open Science Framework (OSF) page (https://osf.io/f6nbu/files/) as protosc_v1.03.zip.

### Image processing methods

Images of different categories can be loaded from multiple directories (with each directory corresponding to a different category), from a single directory (by supplying additional information about filename criteria for each condition). Color images can be converted to grayscale if desired. Otherwise, color images can be dealt with as RGB values or converted to cie l*a*b*. From the 3D image-matrices, users can select which layer(s) they want to include in the analysis (for example, they can choose to only continue with the a* layer or the combination of the R and B layers). The user can choose whether they want the feature values that will be extracted from different layers to be treated independently from each other during feature selection or to link them together during selection. Linking them together means that they are always tested and included or excluded together even though they may receive different weights in a support vector machine (SVM). Next, the selected layers can be converted to the image features of interest.

### Fourier-related feature space

Images can be converted to Fourier magnitude features with or without phase information or phase information only. When extracting Fourier magnitudes without phase, the absolute values of the two-dimensional fast Fourier transform (FFT2.m from MATLAB) of each image are used. The magnitude spectrum is subsequently down-sampled by taking the sum of all values corresponding to a particular spatial frequency and orientation range. Note that this down-sampling serves two purposes: For one, it reduces the total number of features, meaning the analyses requires less processing power. Second, it disrupts the influence of phase information, meaning the contrast values also lose all indirect spatial specificity. As such, the spatial frequency analysis focuses only on global contrast differences between categories. Default settings down-sample the spectrum into 24 equal-sized spatial frequency bands and 16 orientation bands, resulting in 384 sections. Each section then holds the sum of the magnitudes that were within that area. When extracting Fourier magnitudes *with* phase or phase information only, the images are downscaled to 25×25 pixels (using the default settings) before extracting both the phase and the associated magnitude. Phase and magnitude are always linked together during feature selection.

### HOG feature space

HOG values reflect the presence of edges, per orientation, for each location of an image (Dalal & Triggs, 2005). As such, HOG values can capture the structure within an image. The default settings that the toolbox uses creates HOG features for 9 unsigned (meaning dark to light edges and light to dark edges are pooled together) orientations in non-overlapping 10×10 pixel sections of the image. We chose to use a non-overlapping section for simplicity when visualizing results. The default resolution (10×10) was chosen to be able to capture small structures in the images.

### Color distributions feature space

When extracting color distributions, for each layer of the image, the probability of values in that layer for falling within one of 25 bins is estimated. We suggest to first converted the images to cie l*a*b*. Note that with this methods, the linking of features from different layers is meaningless and therefore they will not be linked together in the feature selection analysis irrespective of the settings given.

### Pixel intensities feature space

To avoid an extremely large feature-space when extracting pixel intensities, the images are first down-sampled to 25×25 pixels. Other than that, no transformations are made other than converting the two- or three-dimensional image to a one-dimensional vector required for the feature selection procedure.

### Data splitting for feature selection and cross-validation

The feature selection algorithm estimates feature relevance using *k*-fold cross-validation. Per default, *k* is set to 10. This means that the feature selection algorithm will be repeated *k* times with a different subset of the data (one fold) serving as holdout data that is only used for cross-validation and thus not for feature selection. Only the remaining *k* − 1 folds are used to select features. There, these remaining *k* − 1 folds are pseudo-randomly split into a training set (default 50% of the data that are not used for the hold-out set) and a validation set. This split is pseudo-random since the training data use an equal number of examples for each class. This was implemented to make sure training is unbiased. Note that, to reduce the possibility of overfitting the data to a specific set of examples, a new pseudo-random split into training and validation data is extracted for each step in the procedure where a feature is selected. Note that, for smaller data sets, few data remain as holdout data per fold, making it more difficult to get accurate performance indicators. In that case, an alternative approach can be used where, for each fold, 50% of the data are assigned to the training set and the remaining data are used for the holdout set.

### Univariate feature ranking and correlated features

The first step in the feature selection procedure is to score each feature based on the differences between categories within that feature. The default settings calculate the chi-square statistic using Kruskal–Wallis analysis of variance. Starting with the highest-ranking feature, Protosc, per default, checks which features explain at least 25% of each other’s variance and groups those features together. As with linking features together describe above, this grouping means that they are always tested and included or excluded together even though they may receive different weights in an SVM. Note that including instead of excluding correlated features may not be ideal for decoding performance. However, instead of optimizing decoding performance, Protosc aims at finding and weighing relevant features, and correlated features should therefore be included together since the information they represent likely overlaps.

### Feature selection procedures

The feature selection procedures aim to find the features of the images that have predictive value over the predetermined categories the images belong to. Note that, if more than two categories of images are used, Protosc looks for the features that best separate all categories and does not differentiate between features relevant for one pairwise category comparison versus another. The feature selection algorithm creates four selections of features which are subsequently trained on the training data with cross-validation performance estimated on the holdout data. The four selections are based on a filter method and a wrapper method (Kohavi & John, 1997) as well as two random selections. When using a filter method, we selected the features with a *p*-value lower than 0.01, based on Kruskal–Wallis analysis of variance. However, if no features are selected this way, we first rank the chi-square score and test, within the training data, how many of the top-ranking features result in the highest cross-validated performance within the training data. Note that this approach will always include features with monotonically decreasing chi-square scores. The wrapper approach does not have this restriction. For the wrapper approach, we use a custom-built stepwise inclusion algorithm where inclusion is based on machine learning performance. Details of the wrapper are described in the next paragraph. The algorithm continues until it has selected the same amount of features as the filter selection. Finally, two additional feature sets are suggested as well. These last two sets contain the same number of features, but here the features are selected either randomly or pseudo randomly. Specifically, a “random” selection simply selects a random selection of features for training. A “pseudorandom” selection selects a random collection of the features that are not part of either the current wrapper selection or the current filter selection. These last two selections are included as backup options for when the wrapper and/or filter selection(s) fail to predict the holdout data well, which could, for example, be due to overfitting on the training data or by a too narrow search space. Moreover, performance of the random selection can aid with interpretation of the feature selection performance since high decoding performance based on random selection indicates an easy decoding problem where many features are relevant. The four sets of features are next used to train classification models using all current training and validation data. The classification method used here can be selected by the user (default settings use a linear support vector machine; this classifier does not have to be the same classifier as used for the wrapper feature selection process). Note that the data used for training are again balanced to contain equal representations of each class. The models are then cross-validated on the holdout data. As a reference, performance based on all features, referred to as a full model, is also estimated.

### Wrapper method

**Fig. 1:**
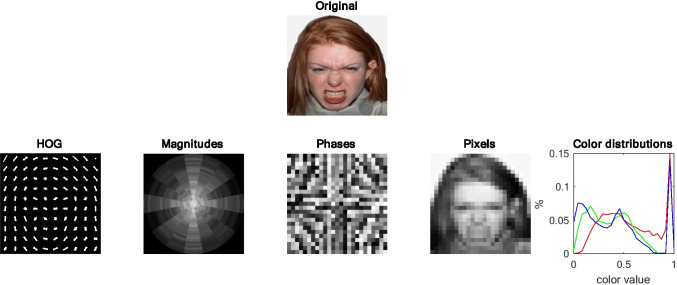
Examples of different features (from left to right: HOG, Fourier magnitudes, Fourier phases, pixel intensities and color value distributions, with the color values on the *x*-axis and the percentages on the *y*-axis) extracted form an example image (top).

**Fig. 2:**
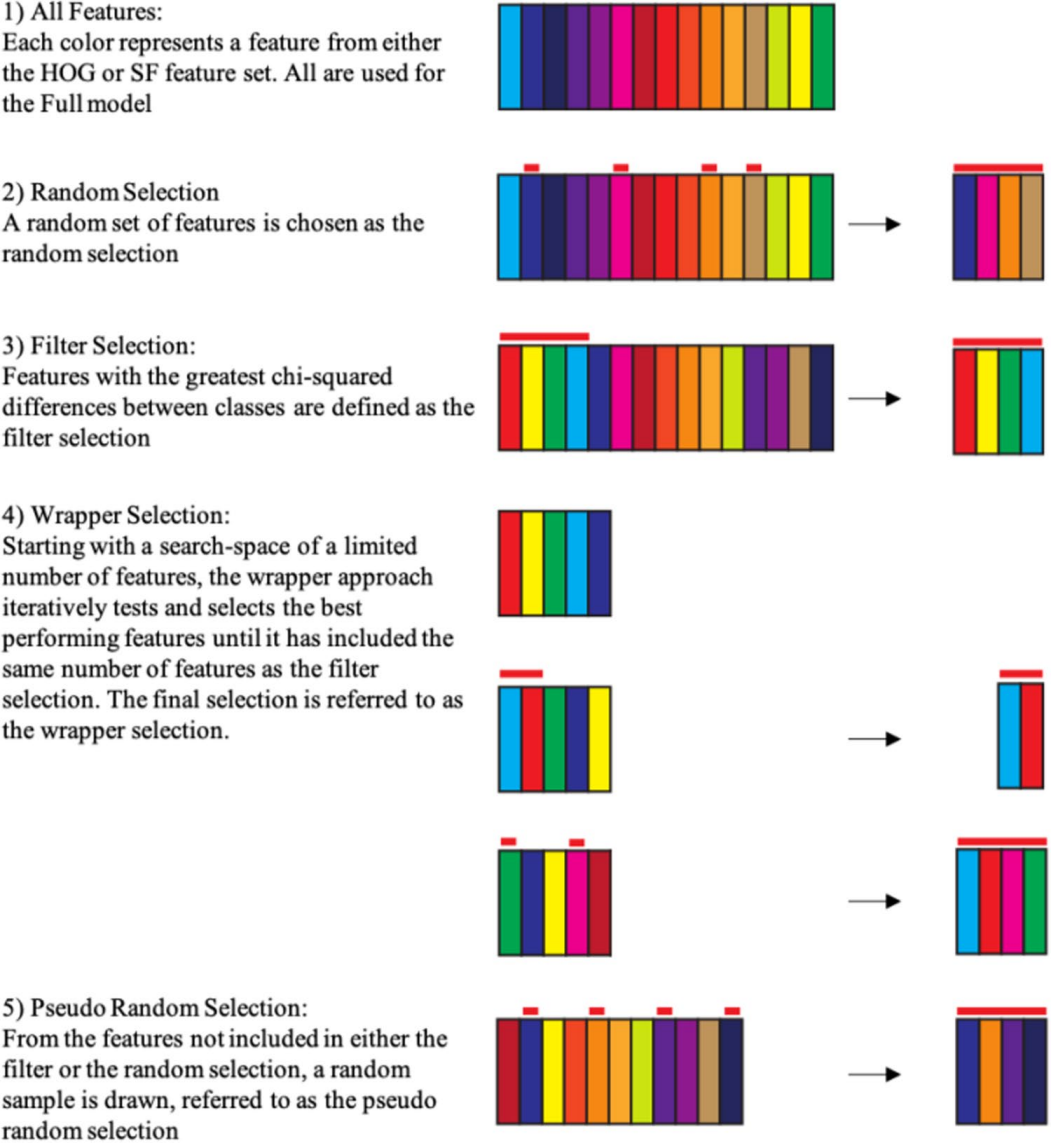
Feature selection methods. Schematic representation of the feature selection methods used in in Protosc. (1) Visual representation of the feature set. As a comparison to feature selection performance, all of the available features will be used to train and test a model referred to as the Full model. (2) A random collection of features (indicated by the red bars) is selected for as the random selection. (3) The features are ranked based on chi-square scores. The top of the ranking is used to determine the filter model. (4) A search space is defined from the top-ranking features and the features in this search space are tested for inclusion into the wrapper selection through an iterative process until enough features have been selected for the wrapper model. (5) From the residual features, unused by the wrapper or filter selections, a random selection is made for a pseudorandom selection.

To take into account potential interactions between features that help separate, and thus decode, image categories, a stepwise feature inclusion algorithm, referred to as a wrapper, is used. The wrapper adds clusters of correlated features until it has selected the same amount of features as the filter selection. To do so, each time the wrapper tries to add features to the selection, it first estimates a reference for performance using the currently selected features by training on the training data and testing on the validation data. If no features are yet selected, one divided by the number of classes is used as a reference performance. Next, using the same training and validation data, it tests the degree to which the addition of a feature (or cluster of features) changes performance. Note that it does not test the addition of all remaining features. The wrapper only searches the n features with the highest rankings. Here, n is referred as the search space size and is, on default, set to two times the maximum amount of features to be included in the selection. For each iteration of the wrapper, this process is repeated four times, and the average change in performance based on the addition of a cluster of correlated features in the search space is calculated. The features that increase performance are sorted in descending order and the top n features are included in the wrapper selection. Here, n is set to 25% of the maximum amount of features to be included. When the size of the wrapper selection first exceeds 75% of the maximum amount of features to be included, each cluster of features is tested for its relevance. Specifically, clusters of features are removed one by one, and if removal results in an increase in performance, it is removed from the wrapper selection Figs. 1 and 2.

### Comparisons to chance performance

To estimate a distribution of chance performance, for each fold and for each method’s selection of features, 25 models are trained and tested based on shuffled category labels. The result is a distribution of *k* times 25 times 4 values forming a distribution of chance performances used for a permutation test. The probability of the performance for each of the four methods of selection is subsequently based on this distribution. However, the main interest is in the features that were associated with low-probability chance performance, not the individual performances associated with these four models. Concerning the individual features, the corresponding null-hypothesis is that there are no features of relevance for separating the classes. To test for features of relevance, we first collect all features associated with performance above the 99th percentile. We choose the 99th percentile to correct for using four methods of selection. Note that not all features are tested an equal number of times. Therefore, to take into account the number of times a feature has been tested and the associated regression to the mean, a feature is determined as relevant when the average performance associated with the feature is higher than the maximum value in control distributions that also takes into account regression to the mean. This means that for features used only once, performance needs to exceed the maximum of the permutation test distribution. However, if the feature has been used, for example, ten times, we create a new control distribution that takes *k* times 25 times four averages of ten values from the original permutation test distribution and the features average should exceed the maximum in that distribution. Note that this means that features that are not consistently chosen have a more conservative criterion. Furthermore, this separation between testing the performances associated with a particular method of feature selection and performance associated with a particular feature makes it possible to find significant features even when a particular method for feature selection fails to reach significance.

## Results

### Analysis 1: Comparison to ground truth and similar methods

To test whether the algorithm actually finds the features of relevance, we created a mock data set of 400 features with two classes and 250 examples per class. Each feature was filled with values drawn from a normal distribution with a mean of 0. Prior to manipulating the data as described next, both classes held the same values per feature but the corresponding values were assigned to different examples. This was done to ensure there were no differences in the distributions per class as a whole. Note that, with splitting the data into training and tests sets during *k*-fold cross-validation, differences in a feature can now arise even when there were none. We next randomly selected 25 features and increased all values for those features in class one. These increases reached from .5 to .25 standard deviations of the distribution in 25 linear steps. Using this mock data set, we applied the Protosc approach to the selected features. For comparison and using the same folding as used with the Protosc approach, we also applied (1) feature selection via the *t*-test approach at multiple criteria (uncorrected for multiple comparisons: *p* < 0.05, *p* < 00.01 and *p* < 00.001, which is the same criterion as Protosc) to suggest significant features and (2) the methods recommend by MATLAB for feature selection (MATLAB's adaptation of neighborhood component analysis with regularization [fscnca]). Feature selection via all methods was repeated 30 times, each with new mock data. The full procedure was repeated for 50 and 100 manipulated features. With the exception of the *t*-test approach at a criterion of *p* < 00.05 in the 25 relevant features condition, Protosc detects the most relevant features even when it uses a more stringent criterion. While all *t*-test versions are robust against false alarms, Protosc does occasionally make a false alarm. The average number of false positives, however, stays below 1. Although fscnca resulted in a very high number of false positives, hyper-tuning of the tolerance for inclusion would have decreased this value. Still, the overall percentage of relevant features detected for the MATLAB approach was relatively low with 52% (Table 1). Protosc had the overall highest detection performance (76%) with the *t*-test approach at the same criterion as Protosc (*p* < 00.001) having the lowest detection performance (44%; Table 1). Taken together, these results show high sensitivity with low false detection for finding relevant features using the Protosc approach.

**Table 1:** Results of analysis 1

	Results analysis 1									
Method	*Protosc (p < 00.001)*		*t-test (p < 00.05)*		*t-test (p < 00.01)*		*t-test (p < 00.001)*		*fscnca (tol=0.25)*	
Metric	*Mean Hits*	*Mean FA*	*Mean Hits*	*Mean FA*	*Mean Hits*	*Mean FA*	*Mean Hits*	*Mean FA*	*Mean Hits*	*Mean FA*
Relevant features: 25	16.97	0.00	18.14	0.00	14.84	0.00	11.12	0.00	14.14	97.93
Relevant features: 50	39.37	0.30	36.50	0.00	29.84	0.00	22.09	0.00	25.91	82.46
Relevant features: 100	81.57	0.03	73.42	0.00	59.79	0.00	44.03	0.00	46.99	56.94
Overall percentage found	M: 76.06% SD: 8.30%		M: 72.99% SD: 1.75%		M: 59.61% SD: 2.05%		M: 44.22% SD: 3.00%		M: 51.78% SD: 5.06%	

The table shows the mean number of hits and mean number of false alarms (FA) per feature selection method and per number of relevant features in the mock data set over 30 iterations. The bottom row shows the overall mean (M) percentage of features found with the corresponding standard deviation (SD). Results indicate high sensitivity with low false detection for finding relevant features using the Protosc approach

### Analysis 2: Example with face images

For a second demonstration of the toolkit, we analyzed faces from the NimStim face set (Tottenham et al., 2009). This face set has images of facial expression with, among others, open and closed mouths. We used the state of the mouth to label the images and used independent HOG and spatial frequency analyses to test whether we can find the features that represent the difference between an open and a closed mouth. Based on the file names, 291 images were included in category 1: open mouth, whereas 282 images were included in category 2: closed mouth. A total of eight different emotional expressions were represented in each category. First, Protosc located the face area using the Viola-Jones algorithm (Viola & Jones, 2001) and took the area annotated as the face plus an additional 20% as our face images. Note that at this point the images have different dimensions, so next it scaled all images to 200×200 pixels using bicubic interpolation. We then ran separate analyses for three features spaces: Fourier magnitudes (based on grayscale version of the images) to test for global contrast differences, color value distributions to test for global color differences and HOG feature to test for spatially specific edge contrast differences. Note that we ran the analyses separately since the weight associated with a specific feature is not independent of the other features it is simultaneously tested with. That means that if one feature space is far superior to another feature type in its decoding accuracy, but both contain relevant features, the weights associated with the inferior feature space may become inflated.

#### Results for face stimuli

**Fig. 3:**
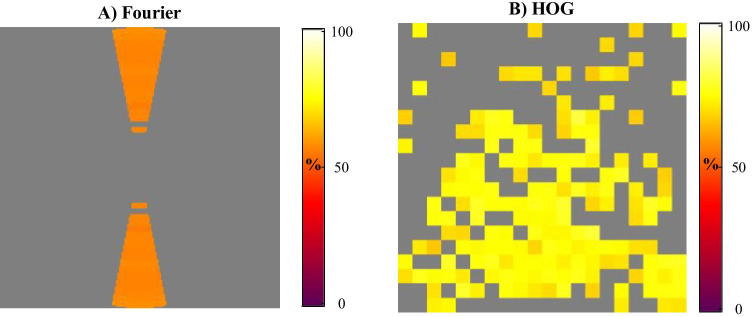
Results analysis 2. Figure 3a visualizes the Fourier magnitude results, 3b the HOG results. Colors reflect the weights of the features, which in turn reflect their average associated machine learning performance (as percentage correct). Figure 3a shows that medium to high spatial frequencies, from horizontal edges in the images, have predictive value concerning the state of the mouth. Figure 3b shows that many local edge contrasts in the images have predictive value over the state of the mouth in the image set used, even outside of the face area, suggesting many of these features may play a confounding role when comparing behavioral responses between open and closed mouthed faces.

Results show 17 features based on Fourier magnitudes, 0 relevant features based on color value distributions, and 172 relevant features based on HOGs. Criterion for relevance was set at *p* < 00.001. Average performance associated with the relevant Fourier features was 57% (SD: 0.7%), and for the HOG feature average performance associated with the relevant features was 74% (SD: 3.1%). The relevant Fourier features are found in the medium to high spatial frequencies reflecting horizontal edges in the images (Fig. 3a). Although the relevant HOG features are found mainly in the lower central part of the image, where we would expect them, many other locations are reflected by the relevant features as well, some even falling outside the face and likely are based on the hair of the persons in the images (Fig. 3b). These latter areas are not of interest when comparing open to closed mouths and the results would at least show that cropping the faces is essential when using this set of images.

## Using the toolbox

The current toolbox was created for a MATLAB environment and is fully compatible with versions 2019 and later. The tool will also work with MATLAB 2017a through 2018b, with the exception that the app will not display tooltips. Protosc requires the Control System Toolbox, Image Processing Toolbox, Statistics and Machine Learning Toolbox, Computer Vision Toolbox, Parallel Computing Toolbox, MATLAB Parallel Serve and Polyspace Bug Finder. Note that the toolbox will also work without the Parallel Computing Toolbox and the MATLAB Parallel Server, just slower. Furthermore, when using MATLAB 2020 and up, MATLAB Parallel Server and Polyspace Bug Finder are not required. All syntax is platform-nonspecific and has been tested on both a PC running Windows 7 and MacBook running OSX Mojave. Protosc can be downloaded from OSF (https://osf.io/f6nbu/) and added to the MATLAB path. The Protosc OSF page further contains a wiki including detailed information about the use of the toolbox, as well as instructional and explanatory videos and a tutorial.

**Fig. 4:**
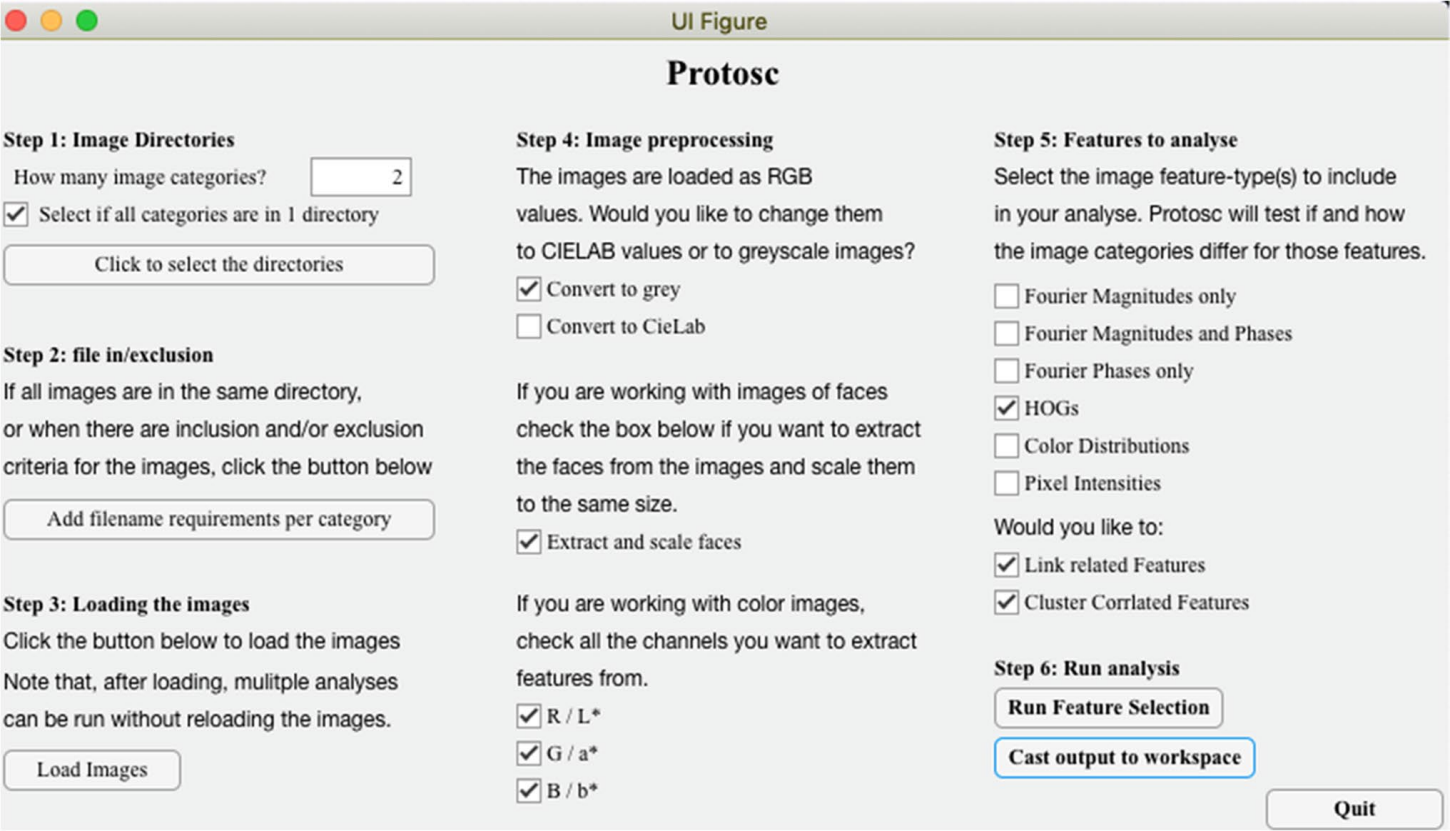
Interface of the Protosc app. The app can be opened via protosc_app.m. The app aids the user to select the images of interest, choose the more basic settings and select an analysis of interest. Alternatively, protosc_template.m creates a new script with a template for the analyses where all settings can be adjusted by the user.

The main method to use the toolbox is via its app (open with protosc_app.m). The interface of the app (Fig. 4) guides the user to select and load image, to choose the basic settings and to run the analyses. Relevant outputs of the analysis are gathered in a struct that can be cast to the MATLAB workspace. Alternatively, protosc_template.m open a creates a new script with a template for the analyses to be adjusted by the user.

### Categories based on behavior

Although the standard functionality of this toolbox is built for decoding predetermined image categories, categorization of the images can also be done by participants in an experiment. For example, the task for a participant may be to indicate whether a neutral face appears more happy or more sad. The experimenter can then place all the images responded to with “more happy” in one directory and all the images responded to with “more sad” in another directory, and test which features best predict the response of the participant. However, creating the two directories per participant is not the most efficient way. To assign behavior-based responses to images and test for features that have predictive value for the given responses, the user can create an Excel file containing one column of numerical labels and one column of filenames and supply it to protosc_ImageFileList_CustomLabels.m. The Excel file should contain numerical labels in column A and filename including the full path of the file in column B. Supplying the Excel file as input to protosc_ImageFileList_CustomLabels creates a file list that can be subsequently used to load the associated images.

Furthermore, the analysis does not need to be based on a singular images. For example, the toolbox was used in Stuit et al. (2021), where participants selected one of two images using an eye movement. Since two images per trial were used, the feature differences for each image pair were calculated and assigned a label that reflected which image the participant selected with an eye movement. A coding example to create feature differences is supplied in the tutorial (found here: https://osf.io/f6nbu/files/) and on the OSF wiki (https://osf.io/f6nbu/wiki/Analyses%20based%20on%20behavior/).

#### After the analysis

To facilitate the user creating an overview of the used methods and gathered results, Protosc supplies functions to automatically write a text file containing a methods section based on the used settings and features included in the analyses via protosc_report_methods.m. Likewise, protosc_report_results.m provides an overview of the results. Finally, when significant feature are found, and the user wants to, for example, use the features as covariates in a subsequent analysis, protosc_report_feature_table writes an .xlsx file with the image filename in column A and the significant features in the subsequent columns.

## Discussion

In this paper, we introduced and outlined the Protosc toolbox. The aim of the toolbox is to allow any researcher using predetermined conditions defined by visual stimuli to find out exactly how their categorized images differ from each other in terms of image features. Specifically, our approach aims to find as many as possible features that have predictive value over the category the image belongs to. Having this information will allow researchers to better understand what may have caused a dependent measure to differ between conditions. Note that finding features that have predicative value concerning their image category does not necessarily mean that an experiment was confounded by stimuli differences. Whether or not something is a confound depends on the interpretation of the results with respect to the used methods, as well as the causal relations between differences between categories and observed behavior. Having a detailed overview of the features can help to avoid confounded conclusions since these differences can be taken into account either in subsequent analyses or in the discussion.

To evaluate the current methodological approach, we first demonstrated the performances on a mock data set with manipulated features. We found that our approach results in a higher percentage of relevant features found relative to comparable methods while maintaining a low false positive rate. However, this toolbox was created with natural images in mind, so naturally we aimed at demonstrating performance on those as well. Note, however, that there is no clear ground truth for which features should be selected by the algorithm. We compared, using the NimStim face set, faces with open mouths to those with closed mouths based on Fourier magnitudes and HOGs. Although the Fourier magnitudes are not spatially specific, the HOGs are spatially specific, meaning that within natural images, centering the object of interest is recommended. There we found differences in global spatial frequency contrasts as well as local edge contrast differences outside of the face relevant for separating the two classes, but these are likely irrelevant for a researcher comparing responses to open and closed mouth faces.

One of the more difficult aspects related to the current feature selection approach is the interpretation of the selected features. More specifically, are they the only features that determine differences between categories? The answer is not very straightforward. Although our first analysis on the mock data set shows that around 70% of the relevant features are consistently detected, we do not know the degree to which this generalizes to natural images due to the lack of a ground truth. The problem here is the immense degrees of freedom in making models with feature selection. To get an exact answer to the question of whether the selected features are most relevant would be nearly impossible. First of all, the number of possible combinations of features is so large that training and testing all possible models would take very, very long. In fact, using only 50 possible features, assuming one second of calculation per model, would take about 6×10^119^ years to calculate. So calculating performance for all possible combinations of features, using images of 200×200 pixels and the default feature extraction settings, would take a number of years that MATLAB calculated as infinite. And honestly, who has that kind of time? However, there is another reason that getting all relevant features is near impossible. The features selected are based on a sample of images representative of its predetermined category. Many more examples of that category are likely to exist. For example, when comparing dogs to cats, one will not use all earthly images of dogs and compare them to all existing images of cats. As such, the selected features will always be biased towards the differences between categories in the training data. With this in mind, our focus has been on optimizing the algorithm to relatively quickly extract a subset of features that proves relevant to separate the categories empirically.

In our approach, each fold is allowed to create models composed of different features relative to the previous fold. This means that, if one of the four feature selection approaches results in significant performance, the images can be decoded via that method, but the features used to achieve that may differ. As such, there is no unified “best” combination of features. For the significance of the features, even though their performances are dependent on the features they are combined with, each one is tested in isolation, so likewise, there is no unified “best” combination. Therefore, the analysis results do not provide insight into which interactions between features best separate the categories. To overcome this, the function protosc_find_feature_combinations compares the performance of a features in combination with another feature, to that of the performance without the second feature. The function does this for each combination of the relevant features and returns a list of feature combinations where the combined performance is larger than two times the standard deviation of all tested combinations.

The current advances in, and popularity of, machine learning makes these methods more and more accessible. Here, we aim to contribute to this availability. However, we also aim to shift some of the focus from high performance to other outputs of such procedures. For that reason, our approach is not so concerned with the absolute performance of the generated classification models. Instead, our focus is on the features that are consistently associated with above-chance classification of two or more categories of images used as conditions in an experiment. We all know that any difference between conditions can be a possible source of a resulting behavioral effect. Increasing our understanding of how exactly two or more conditions differ in terms of their image content should therefore help us understand the source of the effect. As such, this approach should allow researchers to define conditions more freely, meaning with unmanipulated, and thus natural, images.

## Supplementary Information

ESM 1 (DOCX 1448 kb)
