# Supplementary material for: Introducing the Prototypical Stimulus Characteristics Toolbox: Protosc
Source: Behav Res Methods. 2021 Dec 16;54(5):2422–32. doi: 10.3758/s13428-021-01737-9 (PMC9579116; doi:10.3758/s13428-021-01737-9)
Supplement: Supplementary file 1 — (DOCX 1448 kb) [file 13428_2021_1737_MOESM1_ESM.docx]

**Protosc Tutorial**

Tutorial Version 1.0, July 2021

Protosc version v1.03

Table of Contents

[Introduction 2](#_Toc78722546)

[Download and Install 2](#_Toc78722547)

[How to use the App 3](#_Toc78722548)

[For a video on using the app see: https://youtu.be/kkwvvnSzSZ0 3](#_Toc78722549)

[Starting up the app 3](#_Toc78722550)

[Step 1: Image Directories 3](#_Toc78722551)

[Step 2: file in/exclusion 3](#_Toc78722552)

[Step 3: Loading the images 4](#_Toc78722553)

[Step 4: Image preprocessing 4](#_Toc78722554)

[Step 5: Features to analyze 4](#_Toc78722555)

[Step 6: Run analysis 4](#_Toc78722556)

[How to use the autogenerated script 5](#_Toc78722557)

[Setting the directories 5](#_Toc78722558)

[In- and exclusion criteria 5](#_Toc78722559)

[Set the filename for autogenerated methods and results 5](#_Toc78722560)

[Choosing the feature types and additional settings 5](#_Toc78722561)

[Features and Reference Frames 6](#_Toc78722562)

[Analyzing a single feature space 6](#_Toc78722563)

[1) Fourier Magnitudes Only 6](#_Toc78722564)

[2) Fourier Magnitudes and Phases 6](#_Toc78722565)

[3) Fourier Phases Only 7](#_Toc78722566)

[4) HOGs 7](#_Toc78722567)

[5) Color Distributions 7](#_Toc78722568)

[6) Pixel Intensities 8](#_Toc78722569)

[Analyzing multiple feature spaces at once 8](#_Toc78722570)

[Inspecting the data visualizations 9](#_Toc78722571)

[Inspecting the output struct 10](#_Toc78722572)

[Analyses based on behavioral data 11](#_Toc78722573)

[Autogenerated Files 13](#_Toc78722574)

[Examples for customization of analysis 13](#_Toc78722575)

[Directly supplying images without reading them from files 13](#_Toc78722576)

[Adding an aperture to the images in Stims 13](#_Toc78722577)

# Introduction

Studies within the realm of cognitive neuroscience often use different categories of images to define their conditions. For example, a study might be interested in finding differential reaction times to the presentation of faces with a neutral versus an angry facial expression. Since any difference in these categories is a valid candidate to explain category-related behavioral (or neural) differences, knowledge about the objective image differences between categories is crucial for the interpretation of the behaviors (or neural responses). However, natural images vary in many image features, and not every feature is equally important in describing the differences between the categories. Here, we provide a methodological approach for finding as many of the image features as possible that have predictive value over the category the images belong to, using machine learning as a tool. In other words, we describe a means to find the features of a group of images by which the categories can be objectively, and quantitatively, defined. Our method does not aim at providing a means for the best possible decoding performance; instead, our aim is to uncover prototypical characteristics of the categories. To facilitate the use of this method, we offer an open-source, MATLAB-based toolbox that performs such an analysis and aids the user in visualizing the features of relevance. This text provides a guide for installing and using the toolbox.

For an example of application of the beta version of Protosc, see:

[*https://www.nature.com/articles/s41598-021-87881-w#citeas*](about:blank#citeas)

For an application of a previous version of the toolbox, see:

[*https://youtu.be/bN24w16-CZA*](about:blank)

For an image set to try out the toolbox with, see:

*https://www.kaggle.com/chetankv/dogs-cats-images*

# Download and Install

After downloading the .zip file from the OSF files page (https://osf.io/f6nbu/files/), unzip the file. Next, place the Protosc folder in the desired location on your computer. Go to MATLAB “set path” on the Home tab and add the Protosc folder with subfolders (see below). A total of three folders should be added. Then, save the path.

For a video on the installation process see: [https://youtu.be/z23CKgpFJUU](about:blank)


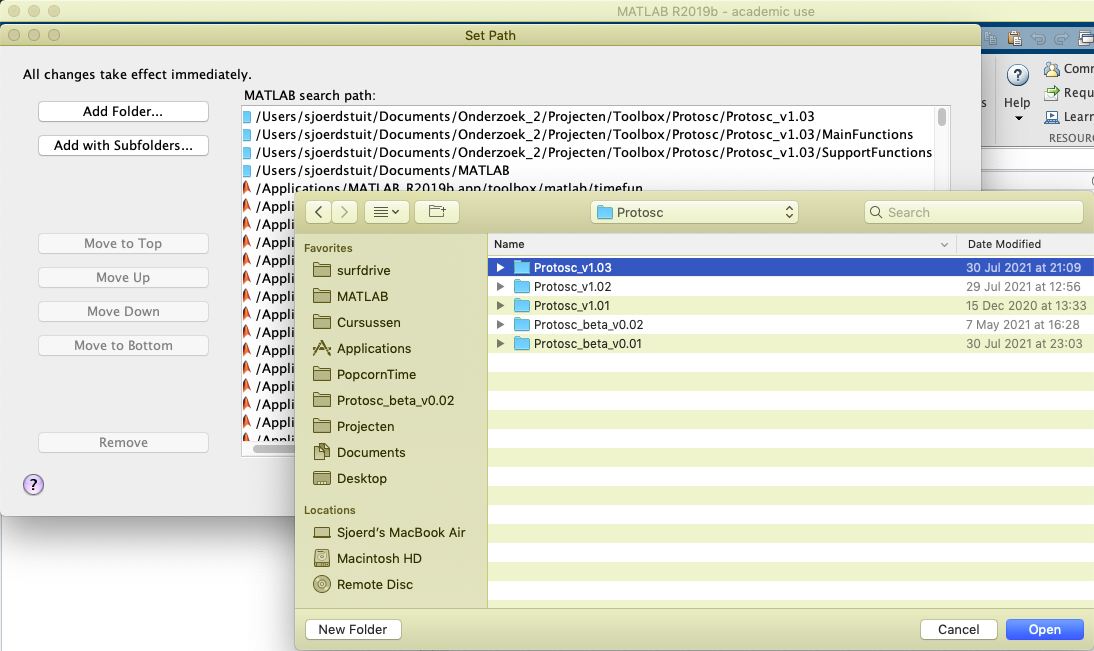


# How to use the App

### For a video on using the app, see: [https://youtu.be/kkwvvnSzSZ0](about:blank)

### Starting up the app

To start the app, run protosc_app.m in the MATLAB command window. The following window will open:


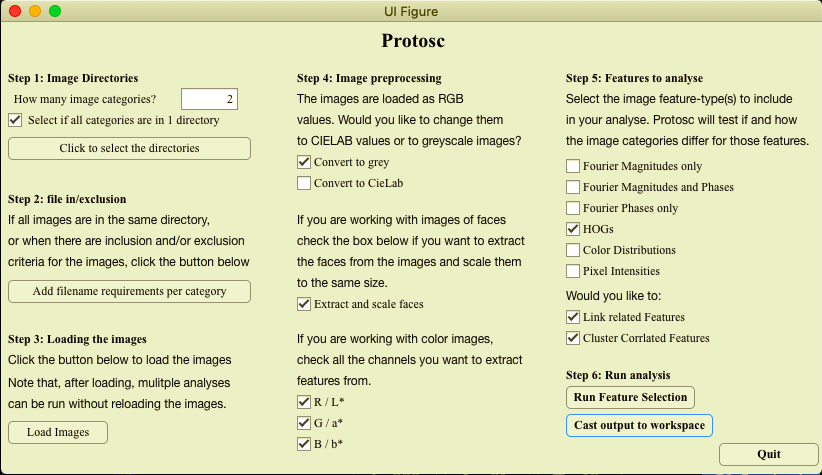


### Step 1: Image Directories

First, indicate how many categories of images you have. When clicking the button under step 1 of the app, one window will open (example below) for each category. However, if you check the box “select if all categories are in 1 directory,” only one window will open. Navigate to the location of the image for category 1 and press “open.” Do the same for the other categories (when applicable).


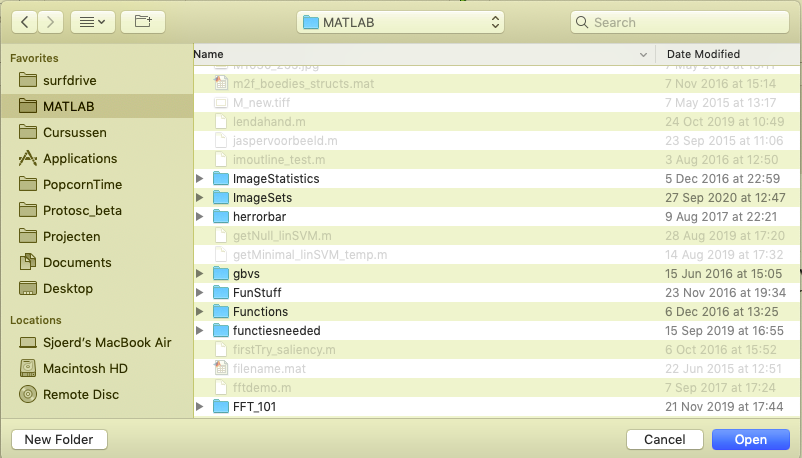


### Step 2: file in/exclusion

This step is required if all images come from a single directory. Otherwise it is optional. Clicking the button in this step will evoke the follow prompt in the MATLAB command window:

*For category 1*

*What should be in the filenames? (please separate multiple terms with * (e.g. term1*term2):*

Here you can indicate what should be part of the filenames for category 1. No quotation marks are required. Multiple filename requirements per category can be given by combining terms with an asterisk (for example: happy*frontal). After answering, Protosc asks if there is anything that should not be in the filenames for category 1. The process is repeated for each category.

### Step 3: Loading the images

After setting up the directories and filename requirements, the image can be loaded. Loading is kept as a separate non-automatized step so that multiple analyses can be run on the same images without reloading them. Note, though, that running the analysis without loading the images will cause the analysis to crash.

### Step 4: Image preprocessing

Here you can choose to convert the images to grayscale images or to the CIELAB color-space. For those working with images of faces, there is the option to extract only the face area from the images and scale all those face areas to images of the same size (see example of extracting the face area in the image below). Note that selecting this option means that only images from which a face can be extracted are selected for the analysis. Finally, you can indicate which channels (for color images) of the images you want to include.

On the left is an example image of a face; on the right is the same face, but now the face area is extracted with the Viola-Jones algorithm. The rest of the image is discarded.


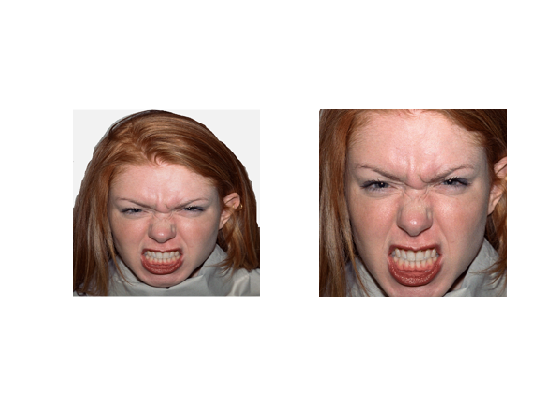


### Step 5: Features to analyze

In step 5 of the app you can indicate which feature types (e.g. HOG Features & Fourier Phases) to include in your analysis. Furthermore, you can indicate whether related features should be linked together and whether correlated features should be clustered (e.g. treated as a single feature). More info on the features, linking and clustering is displayed when placing the cursor over the respective terms.

### Step 6: Run analysis

While running, progress is continuously reported in the MATLAB command window. When complete, the main results are displayed in the command window. To access the output of the analysis, press the cast button under step 6 of the app and the variable *out* will be added to your workspace.

# How to use the autogenerated script

For a video on the autogenerate analysis script, see: [https://youtu.be/7ZfkxZOqmOQ](about:blank)

Running the function protosc_template(filename) will create and open an m-file with a Protosc analysis. Compared to using the app, the script allows direct control over a wide range of settings for the analyses. Also, it is suitable for analyses where the categorical labels of the images are not easily set by the folder they are in, or when they can be separated by their filename.

### Setting the directories

After generating the script, first indicate the location of your images by updating the following line to match your specific location on your computer:

imdir{1} = '/Users/sjoerdstuit/Documents/MATLAB/ImageSets/Nimstim faces/Nimstim faces/';

Note that this example is for when all images are in a single directory. If the images are in separate directories, the variable imdir can be extended as such:

imdir{1} = '/Directory_Category_1/';

imdir{2} = '/Directory_Category_2/';

imdir{n} = '/Directory_Category_n/';

Based on your operating system, different file separators might be required (/ for Mac, \ for Windows).

### Inclusion and exclusion criteria

Next, indicate the file name requirements per category:

mustinclude = {'_O.' '_C.'};

maynotinclude = [];

In this example, filenames for category one must include ‘_O.’ and for category two they must include ‘_C.’. Similarly, filename criteria for exclusion can be given via the variable maynotinclude. Multiple filename requirements per category can be given via an asterisk. For example:

mustinclude = {'_O.*frontal' '_C.*frontal'};

When not having inclusion criteria, simply put

mustinclude = [];

### Set the filename for autogenerated methods and results

After running the analysis, two text files will be created. One describes the methods used for the analysis, the second describes the results of the analysis.

methodsfilename = 'Methods';

resultsfilename = 'Results';

### Choosing the feature types and additional settings

Next, choose the types of features you wish to include in your analysis:

get_FourierFeatures = 1;

get_Fourier_Mag_Pha = 0;

get_Fourier_Pha = 0;

get_HOGFeatures = 0;

get_ColDistributions = 0;

get_Pixels = 0;

Each of the above variables is used as a Boolean to indicate of the corresponding feature type should be included in the analysis. The script continues with a list of the main settings, including a description of each setting. After adjusting the settings, the script is ready and the analysis can be run. The output of the analysis can be found in the variable *out*.

# Features and Reference Frames

Protosc extracts multiple features from a feature space and tests which of those features can be used to separate the categories of images. This section will go through all the available feature spaces in Protosc and will explain how to keep track of what a particular feature represents in an image.

The feature spaces used by Protosc vary in their dimensions. For example, the Fourier magnitude features come from a two-dimensional space, while the color distribution feature comes from a one-dimensional space. Although a feature might be presented in a two-dimensional space, some analyses (e.g. the feature selection algorithm) require each example (image) to be represented by a one-dimensional vector. This means that the feature space is often transformed in the toolbox. To keep track of this transformation, a reference frame is created for each feature space. The extraction of features from images and the reference frame that comes with them is described below for each feature space separately.

## Analyzing a single feature space

### 1) Fourier Magnitudes Only

*The features*

When extracting Fourier magnitudes without information on the phase, the images are resized to 200 by 200 pixels and the absolute values of the 2D FFT (FFT2.m from MATLAB) of each image are extracted. The magnitude spectrum is subsequently down-sampled by taking the sum of all values corresponding to a particular spatial frequency and orientation range. Note that this down-sampling serves two purposes: One, it reduces the total number of features, meaning the analyses require less processing power. Second, it disrupts the influence of phase information, meaning that contrast values also lose all indirect spatial specificity. As such, the spatial frequency analysis focuses only on global contrast differences between categories. Default settings down-sample the spectrum into 24 equal-sized spatial frequency bands and 16 orientation bands, resulting in 384 sections. Each section then holds the sum of the magnitudes that were within that area.

*The reference frame*

For grayscale images, the reference frame consists of a 200×200 matrix containing 384 wedges, one for each spatial frequency and orientation combination. The central point in the reference frame reflects the lowest spatial frequency, with the frequency increasing outwards. Orientation is reflected by the radial angle, with spatial frequencies along the vertical axis reflecting horizontal edges in the images, 45 degrees clockwise from vertical reflects 45 degrees counterclockwise from horizontal edges in the images, and so on. When using color images and without linking the features, the reference frames for each color channel used are concatenated. For example, an analysis with unlinked features and three color channels results in a 200×600 matrix. When linking the features, color channels are stacked in the third dimension.

The function for extracting this feature type and the corresponding reference frame is protosc_Stims2Fourier_Magnitude_Features_Segmented

### 2) Fourier Magnitudes and Phases

*The features*

When extracting Fourier magnitudes *with* phases, the images are downscaled to 25×25 pixels (using the default settings) before extracting both the phase and the associated magnitude to avoid extremely large feature spaces. The phases and magnitudes of a color channel are always linked together during feature selection.

*The reference frame*

For grayscale images, the reference frame consists of a 25×25×2 matrix where each point reflects one spatial frequency and orientation combination. The first channel (the third dimension of the matrix) contains Fourier magnitudes and the second channel contains the corresponding phases. The central point in the reference frame reflects the lowest spatial frequency, with the frequency increasing outwards. Orientation is reflected by the radial angle, with spatial frequencies along the vertical axis reflecting horizontal edges in the images, 45 degrees clockwise from vertical reflects 45 degrees counterclockwise from horizontal edges in the images, and so on. When using color images and without linking the features, the reference frames for each color channel used are concatenated. For example, an analysis with unlinked features and three color channels results in a 25×75×2 matrix. When linking the features, color channels are stacked in the third dimension, resulting in a 25×25×6 matrix.

Function for extracting this feature type and the corresponding reference frame: protosc_Stims2Fourier_Magnitude_Phase_Features

### 3) Fourier Phases Only

*The features*

When extracting Fourier phase information only, the images are also downscaled to 25×25 pixels (using the default settings) before extracting both the phases to avoid extremely large feature spaces.

*The reference frame*

For grayscale images, the reference frame consists of a 25×25 matrix where each point reflects one spatial frequency and orientation combination. The central point in the reference frame reflects the lowest spatial frequency, with the frequency increasing outwards. Orientation is reflected by the radial angle, with spatial frequencies along the vertical axis reflecting horizontal edges in the images, 45 degrees clockwise from vertical reflects 45 degrees counterclockwise from horizontal edges in the images, and so on. When using color images and without linking the features, the reference frames for each color channel used are concatenated. For example, an analysis with unlinked features and three color channels results in a 25×75 matrix. When linking the features, color channels are stacked in the third dimension, resulting in a 25×25×3 matrix.

Function for extracting this feature type and the corresponding reference frame: protosc_Stims2Fourier_Phase_Features

### 4) HOGs

*The features*

HOG values reflect the presence of edges, per orientation, for each location of an image (Dalal & Triggs, 2005). As such, HOG values can capture the structure within an image. Before extracting the HOG features, the images are resized to 200×200 pixels to ensure they are all the same size. The default settings that the toolbox uses creates HOG features for nine unsigned (meaning dark to light edges and light to dark edges are pooled together) orientations in nonoverlapping 10×10 pixel sections of the image. We chose to use nonoverlapping section for simplicity when visualizing results. The default resolution (10×10) was chosen to be able to capture small structural aspects of the images.

*The reference frame*

The reference frame consists of a matrix of 20×20 cells, each cell covering 10×10 pixels of the image, with the same spatial organization as the original image. This means the upper left element in the plane corresponds to HOG features in the upper-left 10×10 pixels in the image. The progression continues downward until the 20th cell, then starts again in the second column.

Function for extracting this feature type and the corresponding reference frame: protosc_Stims2HOG_Features

### 5) Color Distributions

*The features*

When extracting color distributions, the probability of values in that layer for falling within one of 25 bins is estimated for each layer of the image. We suggest to first convert the images to cie l*a*b*. Note that with this method, the linking of features from different layers is meaningless and therefore they will not be linked together in the feature selection analysis irrespective of the settings given.

*The reference frame*

The reference frame consists of a 3×25 matrix where each row reflect a different color channel (from top to bottom: R/L*, G/a*,B/b*) and each column represents a bin. Note that during visualization the matrix is made square.

Function for extracting this feature type and the corresponding reference frame: Protosc_ValueDistributions_Features

### 6) Pixel Intensities

*The features*

To avoid an extremely large feature space when extracting pixel intensities, the images are first down-sampled to 25×25 pixels. Other than that, no transformations are made besides converting the two- or three-dimensional image to a one-dimensional vector required for the feature selection procedure.

*The reference frame*

For grayscale images, the reference frame consists of a 25×25 matrix with the same spatial organization as the original image, meaning the upper left element in the plane corresponds to the upper left pixel in the image. When using color images and without linking the features, the reference frames for each used color channel are concatenated. For example, an analysis with unlinked features and three color channels results in a 25×75 matrix. When linking the features, color channels are stacked in the third dimension, resulting in a 25×25×3 matrix.

Function for extracting this feature type and the corresponding reference frame: protosc_Stims2Pixels_Features

## Analyzing multiple feature spaces at once

When selecting multiple feature spaces to extract features from, and when using all those spaces to find the features whose values have predictive value over the category the image belongs to, Protosc will concatenate the different feature spaces. When using the app or the autogenerate script, and assuming default settings, the order of the features in the data matrix used for feature selection is as follows:

Fourier magnitudes (24 spatial frequency bands × 16 orientation bands = 384 features if linked, otherwise 384 features per color channel), Fourier magnitudes and phases (25 rows × 25 columns = 625 features if linked, otherwise 625 features per color channel), Fourier phases (25 × 25 = 625 features if linked, 625 features per color channel), HOG features (20 rows × 20 columns = 400 features), Color distributions (25 features per color channel) and pixel intensities (25 rows × 25 columns = 625 features if linked, otherwise 625 features per color channel).

Thus, when combining Fourier magnitudes with HOG features and using grayscale images, the first 384 columns in the data matrix and in the variable out.MainFindings.All_feature_Weigths (see Inspecting the output struct) will refer to the Fourier magnitudes and the next 400 columns will refer to the HOG features. The reference frames for the used features will be combined in the visualizations of the features.

Another example, now using color images and unlinked features: When combining Fourier phases and pixel Intensities for RGB images, the first 625 features are the Fourier phases for the R channel, the next 625 features are the Fourier phases for the G channel, followed by 625 features Fourier phases for the B channel. Then 625 pixel intensity features the R channel, 625 pixel intensity features for the G channel, and finally 625 pixel intensity features for the B channel. Six reference frames will be combined: three for the Fourier phases and three for the pixel intensities.

# Inspecting the data visualizations

Protosc visualizes the following information by default: (A) the chi-square values, normalized between 0 and 100, for each feature presented in the corresponding reference frame, (B) the percentage cumulative sum scores of the chi-square values, (C) the cross-validation accuracies per method of feature selection and (D) the significant features and their respective weights presented in the corresponding references frame. See an example based on comparing happy to angry faces of the Nimstim data set in the figure below.

A) gives an overview of the degree of differences, in Chi-Squares normalized between zero and one, between the categories for each feature. Please note that this reflects a statistical comparison between samples of the images; the results for the features most relevant for *predicting* which category an image belongs to may differ.

B) shows, via its steepness, how fast the chi-square scores add up. For example, a relatively flat (diagonal) line indicates all feature hold about the same amount of information while a very fast rise followed by a flat line shows only a few features hold all the information. There is one curve for each fold.

In C), the black dashed line shows the mean chance performance while the red dashed line shows the 95^th^ percentile of the chance distribution. In terms of interpretation, we can see, for this example, that a random selection of features already performs rather well, meaning there are many relevant differences between the categories. However, we also see that the pseudo-random selection does not perform above the 95th percentile of the chance distribution. This suggests that, when the random selection can’t include features already selected by the filter or wrapper selection, decoding does not work. This indicates that the filter or wrapper selection is finding the most relevant features, and few to no relevant features are likely remaining.

In D) only the significant features are shown, with the color reflecting their associated mean performance (the figure shown here is decoding happy versus angry facial expressions).

The visualization described above can be recreated with the function protosc_all_figures.m

As noted in the previous section, when analyzing multiple feature spaces at once, the reference frames for the feature spaces are combined. In the example below, Fourier magnitude features are combined with HOG features. The Fourier reference frame is presented on the left in A and D, HOGs on the right.

# Inspecting the output struct

For a video on the output variable, see: [https://youtu.be/qz2hCvz1IYE](about:blank)

The output and results of the analyses are given in the format of a struct. The standard name for the struct is ‘out’. To facilitate ease of use, the most important information is given in the sub-variable MainFindings, whose content can be displayed in the command window via the command out.MainFindings. The following is an example of the returned output:

**
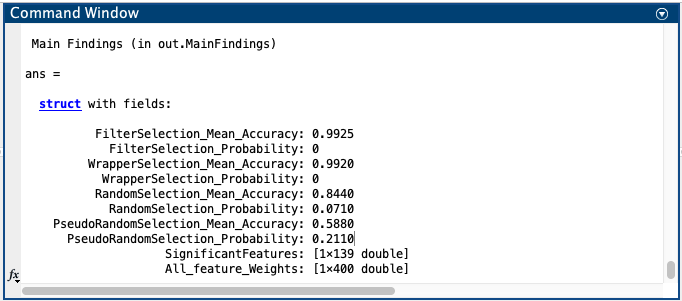
**

The output shows the fraction correct and the associated probability that the accuracy reflects chance performance based on a permutation test of each of the four feature selection procedures of Protosc. Next, it provides a vector of indices of significant features and a vector of the estimated weight of each feature. This example is based on a HOG features using default settings and therefore has a total of 400 features. Well over 25% of the features are estimated to be significant for decoding the classes.

#### Interpreting performance per approach

As noted above, Protosc uses four methods of feature selection simultaneously. Two of those methods, the filter approach and the wrapper approach, are expected to result in significant decoding performance if the categories of images are (easily) separable by their features. The other two approaches, random feature selection and pseudo-random feature selection, are *not* expected to result in significant decoding if there are only a few features that separate the categories by their features. If random feature selection results in significant decoding, the categories can be separated by many different features and the exact combination of features is not very relevant. If pseudo-random feature selection results in significant decoding, it means that there are still many relevant features besides the features selected via the filter and the wrapper approaches.

#### Significant features

The four approach are combined to find features that are reliably associated with above-chance performance. The indices of these features can be found in out.MainFindings.SignificantFeatures. There, the features are given in order of importance (from highest associated decoding performance to lowest). To see where in the reference frame a feature comes from, and thus, what it represents, use the function protosc_find_feature(out,featureindex) and supply the out variable with the feature you want to highlight in the reference frame.

#### All feature weights

The vector out.MainFindings.All_feature_Weigths contains the estimated weight of each feature, irrespective of its significance.

#### The out variable beyond MainFindings

Many aspects of the analysis are stored in the out variable. What follows is a list of relevant variables:

out.AllData contains a matrix of all the data used for the analysis

out.settings contains a struct with all the settings used for the current analysis.

out.codeinfo contains a struct with information concerning the file from which the analysis was ran and date of the analysis.

out.datainfo contains a struct with information on the data used for the analysis such as the number of examples in each category.

out.Results is a nested struct where information on the progress of the analysis is stored.

The most relevant sub-variables are the following:

out.Results.MaxModelParams gives the number of features included per fold of the analysis.

out.Results.Filter, out.Results.Wrapper, out.Results.RandomModel and out.Results. PseudoRandomModel each provide detailed information of the respective feature selection method. Within each of these you can find the F1 score, the accuracies and used features per fold.

# Analyses based on behavioral data

Although the standard functionality of this toolbox is built for decoding predetermined image categories, categorization of the images can also be done by participants in an experiment. For example, the task for a participant may be to indicate whether a neutral face appears more happy or more sad. The experimenter can then place all the images responded to with “more happy” in one directory and all the images responded to with “more sad” in another directory and test which features best predict the response of the participant. However, creating the two directories per participant is not the most efficient way. To assign behavior-based responses to images and test for features that have predictive value for the given responses, the user can create an Excel file containing one column of numerical labels and one column of filenames and supply it to protosc_ImageFileList_CustomLabels.m.

The Excel file should contain numerical labels in column A and filename including the full path of the file in column B. See an example below (the example below is from the file Example_Behavioral_Data.xlsx given with the toolbox).


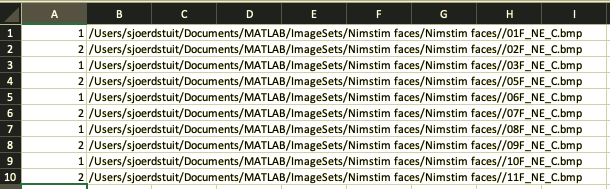


Supplying the Excel file as input to protosc_ImageFileList_CustomLabels creates a file list that can be subsequently used to load the associated images. See an example below.


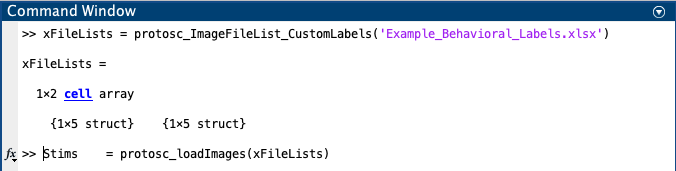


Furthermore, the analysis does not need to be based on a single image. For example, the toolbox was used in Stuit et al. (2021), where participants selected one of two images with an eye movement. Since two images per trial were used, the feature differences for each image pair were calculated and assigned a label that reflected which image the participant selected with an eye movement. The following is an example to create feature differences based on two images:

% Example image feature differences:

% Create settings variable

settings = protosc_Settings;

% Get filelist for images on the left

FileLists_1 = protosc_ImageFileList_CustomLabels(xlsFilename_1);

% load the images

[Stims_1,settings] = protosc_loadImages(FileLists_1,settings);

% convert the images to features

[FourierFeatures_1,refGrid_FourierFeatures] = protosc_Stims2Fourier_Magnitude_Features_Segmented(Stims_1,settings);

% Get filelist for images on the right

FileLists_2 = protosc_ImageFileList_CustomLabels(xlsFilename_2);

% load the images

[Stims_2,settings] = protosc_loadImages(FileLists_2,settings);

% convert the images to features

[FourierFeatures_2,~] = protosc_Stims2Fourier_Magnitude_Features_Segmented(Stims_2,settings);

% subtract one from the other per category (since the features are in a

% cell-type variable)

for category = 1:size(FourierFeatures_1,2)

FourierFeatures_differences{category} = FourierFeatures_1{category} - FourierFeatures_2{category};

end

AllData = protosc_Features2DataMatrix(settings,FourierFeatures);

settings.ReferenceMap = protosc_SetRefGrids(settings,refGrid_FourierFeatures);

out = protosc_FeatureSelection(AllData,settings);

# Autogenerated Files

Protosc can generate three files after running the analyses. The first file is an Excel file containing the features values for the significant features associated with each of the images used in the analysis so they can be used as covariates in subsequent analysis outside of Protosc. The file is generated via: protosc_report_feature_table(out,filename)

The second file is a text file that contains a report on the feature extraction approaches used and the settings for the feature selection analysis. The file is generated via:

protosc_report_methods(out,filename,get_FourierFeatures,get_Fourier_Mag_Pha,get_Fourier_Pha,get_HOGFeatures,get_ColDistributions,get_Pixels)

Note that all inputs after filename are Booleans reflecting the features used in the analysis.

The last file is a text file reporting the statistical results of the analysis (e.g. feature significance and model performance comparisons), including basic interpretations. The file is generated via: protosc_report_results(out,filename)

# Examples for customization of analysis

## Directly supplying images without reading them from files

% The stims variable which contains the images needs a cell for each

% category and in each cell the

% images are placed as follows:

% Stims{category}(example_number).im = the example image

% Here is an example to fill the Stims variable yourself with grayscale

% 100x100 white noise images:

for category = 1:2

for image_number = 1:10

Stim{category}(image_number).im = rand(100);

end

end

## Adding an aperture to the images in Stims

% The following creates an oval aperture with a smooth edge

% width is set a 75% of the image width, height is left at full height

oval = protosc_im_smoothedge_oval(round(size(Stims{1}(1).im,2)*.75),size(Stims{1}(1).im,1));

% go through all categories in Stims

for category = 1:size(Stims,2)

% go through all examples in the category

for example = 1:size(Stims{category},2)

% go through all channels in the image

for channel = 1:size(Stims{category}(example).im,3)

% create an updated version of the Stims variable.

updated_Stims{category}(example).im(:,:,channel) = Stims{category}(example).im(:,:,channel).*oval;

end

end

end

The figure below shows the one of the original images on the left, and the same image but updated with the oval on the right:


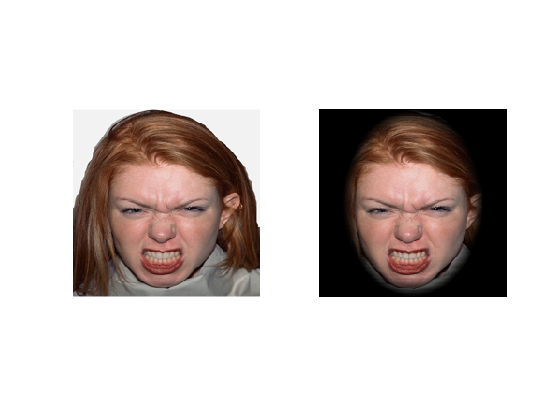


Other functions for creating an aperture are:

protosc_im_circle (for a circular aperture)

protosc_im_smoothedge_circle for a circular aperture with a smooth edge
